# Author Correction: Charge-generating mid-gap trap states define the thermodynamic limit of organic photovoltaic devices

**DOI:** 10.1038/s41467-020-20626-x

**Published:** 2021-01-04

**Authors:** Nasim Zarrabi, Oskar J. Sandberg, Stefan Zeiske, Wei Li, Drew B. Riley, Paul Meredith, Ardalan Armin

**Affiliations:** Sustainable Advanced Materials Programme (Sêr SAM), Department of Physics, Swansea University, Singleton Park, Swansea, SA2 8PP United Kingdom

**Keywords:** Solar cells, Semiconductors, Organic molecules in materials science

Correction to: *Nature Communications* 10.1038/s41467-020-19434-0, published online 04 November 2020.

The original version of the Supplementary Information associated with this Article contained an error in Supplementary Fig. 8 in which the units on the *y*-axes were incorrectly labelled as ‘%’, instead of ‘a.u.’. The HTML has been updated to include a corrected version of the Supplementary Information.

## Supplementary information

Supplementary Information

