# Supplementary material for: Author Correction: Charge-generating mid-gap trap states define the thermodynamic limit of organic photovoltaic devices
Source: Nat Commun. 2021 Jan 4;12:207. doi: 10.1038/s41467-020-20626-x (PMC7782746; doi:10.1038/s41467-020-20626-x)
Supplement: Supplementary file 1 — Supplementary Information [file 41467_2020_20626_MOESM1_ESM.pdf]

Supplementary Information

Charge-generating Mid-gap Trap States Define the Thermodynamic Limit of Organic Photovoltaic Devices

Nasim Zarrabi, Oskar J. Sandberg*, Stefan Zeiske, Wei Li, Drew B. Riley, Paul Meredith and Ardalan Armin*

Sustainable Advanced Materials Program (Sêr SAM), Department of Physics, Swansea University, Singleton Park, Swansea SA2 8PP, United Kingdom

email: o.j.sandberg@swansea.ac.uk; ardalan.armin@swansea.ac.uk

Supplementary Methods

Materials: **PEDOT:PSS** was purchased from Heraeus. Zinc acetate dehydrate, **PCDTBT** (Poly[N-9”-heptadecanyl-2,7-carbazole-alt-5,5-(4’ ,7’ -di-2-thienyl-2’ ,1’ ,3’ -benzothiadiazole)]), **PCPDTBT** (Poly[2,6-(4,4-bis-(2-ethylhexyl)-4H-cyclopenta[2,1-b;3,4-b’)-dithiophene)-alt-4,7 -(2,1,3-benzothiadiazole)]) and **O-IDTBR** were purchased from Sigma Aldrich. **$PC_{70}BM$** ([6,6]-Phenyl-C71-butyric acid methyl ester), **PDINO** (perylene diimide functionalized with amino *N*-oxide) and **EH-IDTBR** were purchased from Solarmer (Beijing). **BQR** (benzodithiophene-quaterthiophene-rhodanine) was provided by Dr. David. J Jones (University of Melbourne). **m-MTDATA** (4,4',4"-Tris[(3-methylphenyl) phenylamino]triphenylamine) was purchased from Ossila. **PM6** (Poly[(2,6-(4,8-bis(5-(2-ethylhexyl-3-fluoro)thiophen-2-yl)-benzo[1,2-b:4,5-b’]dithiophene))-alt-(5,5-(1’,3’-di-2-thienyl-5’,7’-bis(2-ethylhexyl)benzo[1’,2’-c:4’,5’-c’]dithiophene-4,8-dione)]), **Y6** (2,2'-((2Z,2'Z)-((12,13-bis(2-ethylhexyl)-3,9-diundecyl-12,13-dihydro-[1,2,5]thiadiazolo[3,4-e]thieno[2",3’':4’,5']thieno[2',3':4,5]pyrrolo[3,2-g]thieno[2',3':4,5]thieno[3,2-b]indole-2,10-diyl)bis(methanylylidene))bis(5,6-difluoro-3-oxo-2,3-dihydro-1H-indene-2,1-diylidene))dimalononitrile), **ITIC** (3,9-bis(2-methylene-(3-(1,1-dicyanomethylene)-indanone))-5,5,11,11-tetrakis(4-hexylphenyl)-dithieno[2,3-d:2’,3’-d’]-s-indaceno[1,2-b:5,6-b’]dithiophene), **PBDB-T**(Poly[(2,6-(4,8-bis(5-(2-ethylhexyl)thiophen-2-yl)-benzo[1,2-b:4,5-b’]dithiophene))-alt-(5,5-(1’,3’-di-2-thienyl-5’,7’-bis(2-ethylhexyl)benzo[1’,2’-c:4’,5’-c’]dithiophene-4,8-dione)]) and **PTB7-Th** (Poly[4,8-bis(5-(2-ethylhexyl)thiophen-2-yl)benzo[1,2-b;4,5-b']dithiophene-2,6-diyl-alt-(4-(2-ethylhexyl)-3-fluorothieno[3,4-b]thiophene-)-2-carboxylate-2-6-diyl)]) were purchased from Zhi-yan (Nanjing) Inc.

Substrate preparation: Commercial patterned ITO coated glass substrates from Ossila were used for all devices in this work. All the substrates were cleaned in an Alconox (detergent) aqueous solution bath at 60 °C, followed by sequential sonication in deionize (DI) water, acetone and 2-propanol for 10 minutes each. The cleaned substrates were dried with nitrogen and then treated in UV-Ozone cleaner (Ossila, L2002A2-UK).

Electron/Hole Transport Layer (ETL/HTL) deposition: Solar cells were fabricated with either a conventional or inverted architecture. For the conventional devices, PEDOT:PSS was used as the HTL. A PEDOT:PSS solution was first filtered through a 0.45 μm PVDF filter, then it was spin-coated (6000 rpm for 30s resulting in a thickness of 30 nm) onto ITO substrates

and annealed at 155 °C for 15 minutes. For the inverted devices, ZnO was used as the ETL. A ZnO solution was prepared by dissolving 200 mg of zinc acetate dihydrate in 2-methoxyethanol (2ml) and ethanolamine (56µl). The solution was stirred overnight under ambient conditions and was spin-coated onto ITO substrates (4000 rpm resulting in a thickness of approximately 30 nm). The substrates were annealed at 200 °C for 60 minutes.

Active layer and top electrode deposition

The deposition methods of the active layers are described below for each sample. All top electrodes were deposited by thermal evaporation under a vacuum of 10^{-6} Tor with an appropriate mask (from Ossila) to define a 0.04 cm^2cell area for each Pixel.

BQR:$PC_{70}BM$ devices were fabricated with a conventional architecture (ITO/PEDOT:PSS/BQR:$PC_{70}BM$/Ca/Al). For as cast devices, BQR and $PC_{70}BM$ were dissolved in toluene (24 mg/ml with the donor:acceptor ratio of 1:1) and stirred at 60 °C for 3 hours. Then BQR:$PC_{70}BM$ solution was spin coated (1000 rpm) on the PEDOT:PSS layer to achieve a film thickness of 100 nm. For solvent annealed (SVA) devices, the BQR:$PC_{70}BM$ films were further exposed to a Tetrahydrofuran (THF) environment in a closed petri dish for 20s and then thermally annealed (90 °C) for 10 mins. For both SVA and as cast devices, 20 nm of calcium (Ca) and 100 nm of Aluminium (Al) were evaporated as the top electrodes.

PCDTBT:$PC_{70}BM$:m-MTDATA devices were fabricated with an inverted architecture (ITO/ZnO/PCDTBT:$PC_{70}BM$:m-MTDATA/MoO_3/Ag). 30 mg of PCDTBT:$PC_{70}BM$ with a blend ratio of 1:4 (i.e. 6 mg of PCDTBT and 24 mg of $PC_{70}BM$) was firstly dissolved in 800 ul of Chlorobenzene (CB) (3 batches). 200 ul of a solution containing 0.06 mg, 0.006 mg, and 0 mg of m-MTDATA (Mw=789.02 g/mol) was then added to the first solutions in order to have the final solutions containing 1%, 0.1% and 0% by weight of m-MTDATA in PCDTBT. The solution was spin-coated using a spin rate of 800 rpm to obtain an active layer thickness of 90 nm. 7 nm of MoO_3 and 100 nm of Ag were then evaporated as the top electrode.

PCDTBT:$PC_{70}BM$ devices were fabricated with a conventional architecture (ITO/PEDOT:PSS/PCDTBT:$PC_{70}BM$/PDINO/Ag). PCDTBT and $PC_{70}BM$ were dissolved in Dichlorobenzene (DCB) with the donor:acceptor ratio of 1:4, and the thicknesses of the active layers were adjusted by changing the concentration of the solution and the speed of spin-coating (30 mg ml^{-1} DCB solution with 1500 rpm for 54 nm active layer, 40 mg ml^{-1} DCB solution with 1500 rpm for 85 nm active layer, 40 mg ml^{-1} DCB solution with 1000 rpm for 105 nm active layer, 40 mg ml^{-1} DCB solution with 600 rpm for 155 nm active layer, 50 mg ml^{-1} DCB solution with 600 rpm for 185 nm active layer, 60 mg ml^{-1} DCB solution with 600 rpm for 315 nm active layer, 60 mg ml^{-1} DCB solution with 400 rpm for 585 nm active layer). 10 nm of PDINO was cast on the active layer from a methanol solution (1 mg ml^{-1}), then 100 nm of Ag was deposited on the PDINO to form a cathode.

PM6:Y6 devices were fabricated with an inverted architecture (ITO/ZnO/PM6:Y6/MoO_3/Ag). PM6:Y6 was dissolved in a CF solution (14 mg ml^{-1} with 0.5 vol.% CN) with a donor:acceptor ratio of 1:1.2, and spin-coated (3000 rpm) on ZnO to form 100 nm film. The cast active layers were further treated with thermal annealing at 110 °C for 10 min. 7 nm of MoO_3 and 100 nm of Ag were evaporated as the top electrode.

PM6:ITIC devices were fabricated with an inverted architecture (ITO/ZnO/PM6:ITIC/MoO_3/Ag). PM6:ITIC was dissolved in a CB solution (18 mg ml^{-1} with 0.5 vol.% DIO) with a donor:acceptor ratio of 1:1, and spin-coated (1000 rpm) on ZnO to form

100 nm film. The active layers were further treated with thermal annealing at 100 °C for 10 min. 7 nm of MoO_3 and 100 nm of Ag were evaporated as the top electrode.

PM6:O-IDTBR devices were fabricated with an inverted architecture (ITO/ZnO/PM6:O-IDTBR/ MoO_3/Ag). PM6:O-IDTBR was dissolved in a CB solution (18 mg ml^{-1}) with a donor:acceptor ratio of 1:1, and spin-coated (1000 rpm) on ZnO to form 100 nm film. 7 nm of MoO_3 and 100 nm of Ag were evaporated as the top electrode.

PBDB-T:EH-IDTBR devices were fabricated with an inverted architecture (ITO/ZnO/PBDB-T:EH-IDTBR/ MoO_3/Ag). PBDB-T:EH-IDTBR was dissolved in a CB solution (14 mg ml^{-1}) with a donor:acceptor ratio of 1:1, and spin-coated (800 rpm) on ZnO to form a 100 nm film. 7 nm of MoO_3 and 100 nm of Ag were evaporated as the top electrode.

PBDB-T:ITIC devices were fabricated with an inverted architecture (ITO/ZnO/PBDB-T:ITIC/MoO_3/Ag). PBDB-T: ITIC was dissolved in a CB solution (14 mg ml^{-1} with 0.5 vol.% DIO) with a donor:acceptor ratio of 1:1, and spin-coated (800 rpm) on ZnO to form 100 nm film. The active layers were further treated with thermal annealing at 100 °C for 10 min. 7 nm of MoO_3 and 100 nm of Ag were evaporated as the top electrode.

PTB7-Th:ITIC devices were fabricated with an inverted architecture (ITO/ZnO/PTB7-Th:ITIC/MoO_3/Ag). PTB7-Th:ITIC was dissolved in a CB solution (14 mg ml^{-1} with 1 vol.% DIO) with a donor:acceptor ratio of 1:1.4, and spin-coated (1000 rpm) on ZnO to form 100 nm film. 7 nm of MoO_3 and 100 nm of Ag were evaporated as the top electrode.

PBDB-T:$PC_{70}BM$ devices were fabricated with an inverted architecture (ITO/ZnO/PBDB-T:$PC_{70}BM$/MoO_3/Ag). PBDB-T:$PC_{70}BM$ was dissolved in a CB solution (14 mg ml^{-1} with 3 vol.% DIO) with a donor:acceptor ratio of 1:1.4, and spin-coated (1000 rpm) on ZnO to form 100 nm film. Then the as-cast films were rinsed with 80 μL of methanol at 4000 rpm for 20 s to remove the residual DIO. 7 nm of MoO_3 and 100 nm of Ag were evaporated as the top electrode.

PTB7-Th:$PC_{70}BM$ devices were fabricated with an inverted architecture (ITO/ZnO/PTB7-Th:$PC_{70}BM$/ MoO_3/Ag). PTB7-Th:$PC_{70}BM$ was dissolved in a CB solution (14 mg ml^{-1} with 3 vol.% DIO) with a donor:acceptor ratio of 1:1.5, and spin-coated (600 rpm) on ZnO to form 100 nm film. Then the as-cast films were rinsed with 80 μL of methanol at 4000 rpm for 20 s to remove the residual DIO. 7 nm of MoO_3 and 100 nm of Ag were evaporated as the top electrode.

PCPDTBT:$PC_{70}BM$: PCPDTBT:$PC_{70}BM$ devices were fabricated with an inverted architecture (ITO/ZnO/PCDTBT:$PC_{70}BM$/MoO_3/Ag). PCPDTBT:$PC_{70}BM$ was dissolved in a DCB solution (40 mg ml^{-1}) with a donor:acceptor ratio of 1:4, and spin-coated (1500 rpm) on ZnO to form 80 nm film. 7 nm of MoO_3 and 100 nm of Ag were evaporated as the top electrode.

Crystalline silicon solar cell: Commercial crystalline silicon solar cell (Part number: KXOB22-12X1).
Germanium Photodiode: Purchased from Newport (818-IR)

(a)

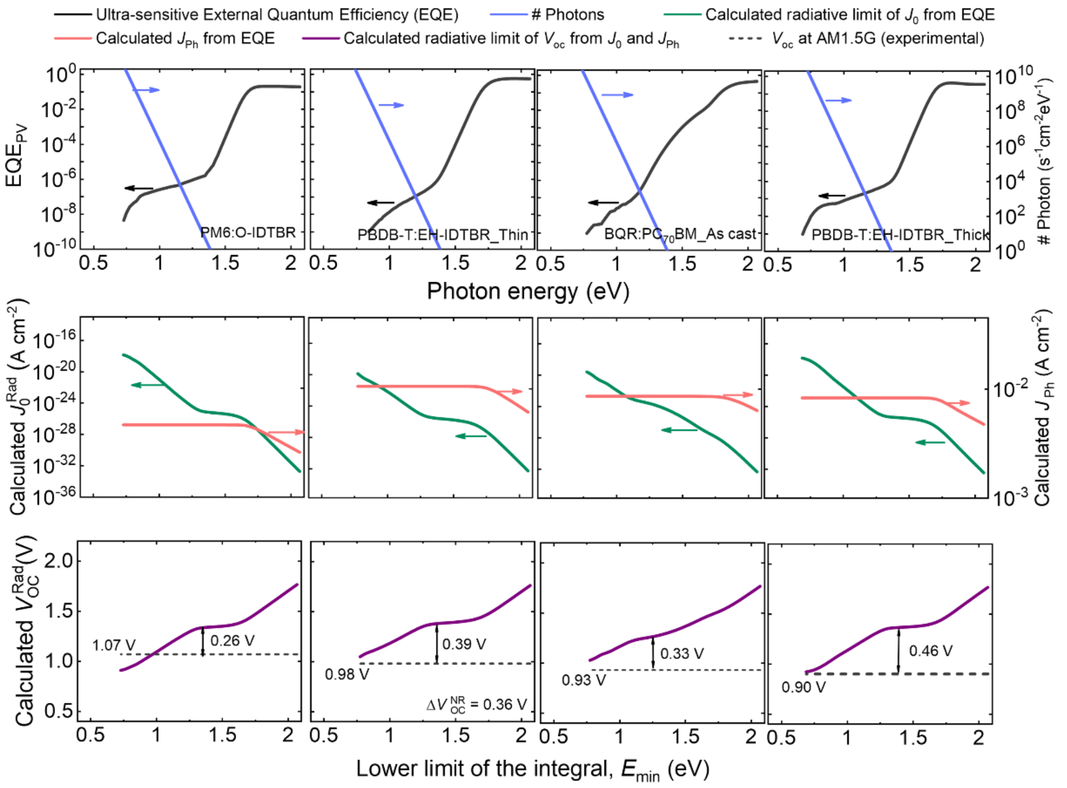
Ultra-sensitive External Quantum Efficiency (EQE)
Photons
Calculated radiative limit of J_0 from EQE
Calculated J_{Ph} from EQE
Calculated radiative limit of V_{oc} from J_0 and J_{Ph}
V_{oc} at AM1.5G (experimental)
EQE_{PV}
Photon ($s^{-1}cm^{-2}eV^{-1}$)
PM6:O-IDTBR
PBDB-T:EH-IDTBR_Thin
BQR:PC_{70}BM_As cast
PBDB-T:EH-IDTBR_Thick
Photon energy (eV)
Calculated J_0^{Rad} (A cm^{-2})
Calculated J_{Ph} (A cm^{-2})
Calculated V_{OC}^{Rad} (V)
1.07 V
0.26 V
0.98 V
0.39 V
ΔV_{OC}^{NR} = 0.36 V
0.93 V
0.33 V
0.90 V
0.46 V
Lower limit of the integral, E_{min} (eV)

(b)

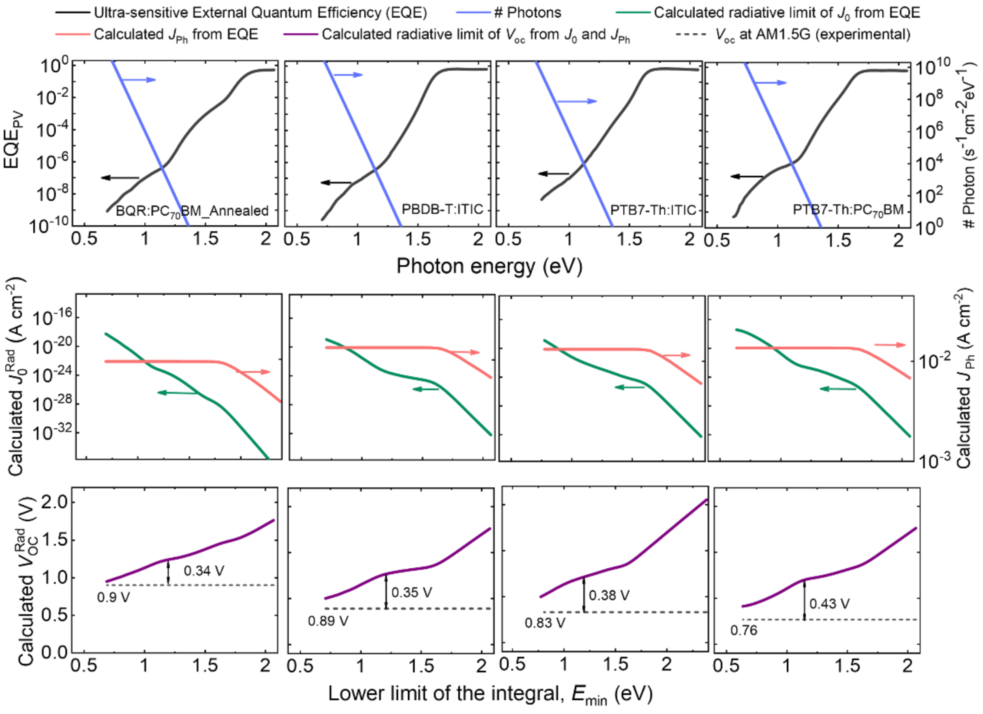
Ultra-sensitive External Quantum Efficiency (EQE)
Photons
Calculated radiative limit of J_0 from EQE
Calculated J_{Ph} from EQE
Calculated radiative limit of V_{oc} from J_0 and J_{Ph}
V_{oc} at AM1.5G (experimental)
EQE_{PV}
Photon ($s^{-1}cm^{-2}eV^{-1}$)
BQR:PC_{70}BM_Annealed
PBDB-T:ITIC
PTB7-Th:ITIC
PTB7-Th:PC_{70}BM
Photon energy (eV)
Calculated J_0^{Rad} (A cm^{-2})
Calculated J_{Ph} (A cm^{-2})
Calculated V_{OC}^{Rad} (V)
0.9 V
0.34 V
0.89 V
0.35 V
0.83 V
0.38 V
0.76
0.43 V
Lower limit of the integral, E_{min} (eV)

(c)

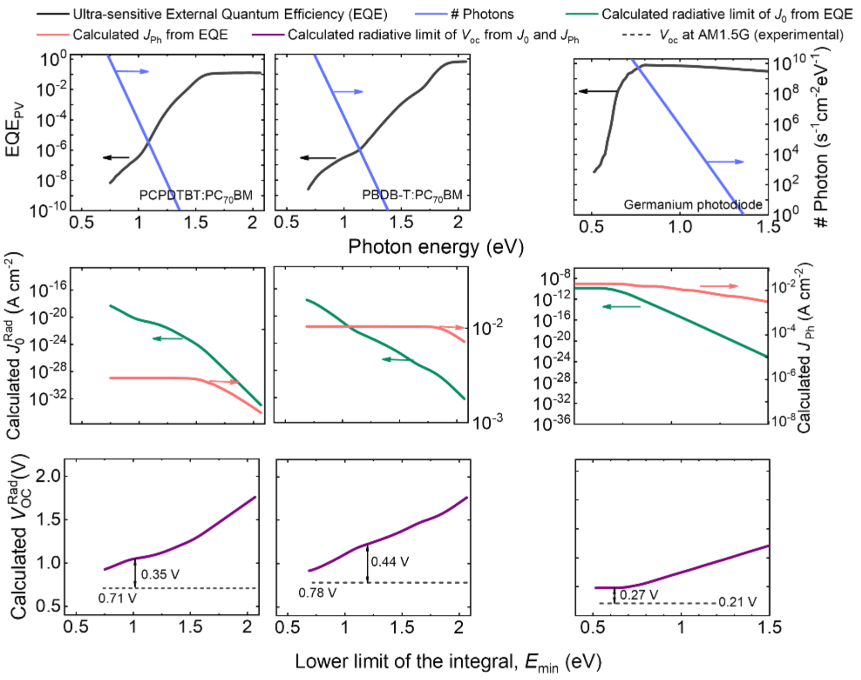

Supplementary Figure 1. (a), (b) and (c) Experimental $\mathrm{EQE_{PV}}$ and the calculated, J_0^{Rad}, J_{Ph}, $V_{\mathrm{OC}}^{\mathrm{Rad}}$ versus photon energy. Experimental $\mathrm{EQE_{PV}}$ and the calculated, J_0^{Rad}, J_{Ph}, $V_{\mathrm{OC}}^{\mathrm{Rad}}$ versus photon energy. In the upper panel, the black curve represents the experimental $\mathrm{EQE_{PV}}$ *versus* photon energy for 4 different solar cells. The limit of the sensitive $\mathrm{EQE_{PV}}$ (reported before) and the ultra-sensitive $\mathrm{EQE_{PV}}$ (reported in this work) are shown in the first plot with dotted lines. The corresponding Φ_{BB} *versus* photon energy is plotted on the right axis (blue curve). Ultra-sensitive $\mathrm{EQE_{PV}}$ measurements reveal sub-gap features in the $\mathrm{EQE_{PV}}$ spectrum. In the middle panel the calculated $J_0^{\mathrm{Rad}}(E_{\mathrm{min}}) = q\int_{E_{\mathrm{min}}}^{\infty} \mathrm{EQE_{PV}}\Phi_{\mathrm{BB}}\, dE$ (the green curve on the left axis) and $J_{\mathrm{Ph}}(E_{\mathrm{min}}) = q\int_{E_{\mathrm{min}}}^{\infty} \mathrm{EQE_{PV}}\Phi_{\mathrm{sun}}\, dE$ (the pink curve on the right axis) are shown *versus* the photon energy. For comparison, the CT state energy (green) and optical gap (pink) have been included as indicated by the vertical dashed lines. In the lower panel, the calculated $V_{\mathrm{OC}}^{\mathrm{Rad}}(E_{\mathrm{min}})$ (solid purple curve) as a function of the photon energy and the experimental V_{OC} measured at 1 sun illumination (dashed lines) are shown. The corresponding $\Delta V_{\mathrm{OC}}^{\mathrm{NR}}$, calculated from the measured $\mathrm{EQE_{LED}}$ using $q\Delta V_{\mathrm{OC}}^{\mathrm{NR}} = -kT\ln(\mathrm{EQE_{LED}})$, are shown as legends.

Supplementary Note 1: Details of the Gaussian fits:

For the ultra-sensitive $\mathrm{EQE_{PV}}$ fittings, the following expression, in accordance with Equations 2 and 3 in the main text, were used to fit the sub-gap features:

$$\mathrm{EQE_{PV}}(E) = \frac{f_{\mathrm{CT}}}{E\sqrt{4\pi\lambda_{CT}kT}} \exp\left(-\frac{[E_{\mathrm{CT}} + \lambda_{\mathrm{CT}} - E]^2}{4\lambda_{\mathrm{CT}}kT}\right) + \frac{f_{\mathrm{t}}}{E\sqrt{4\pi\lambda_{\mathrm{t}}kT}} \exp\left(-\frac{[E_{\mathrm{t}} + \lambda_{\mathrm{t}} - E]^2}{4\lambda_{\mathrm{t}}kT}\right) \quad (1)$$

where on the right-hand side the first term corresponds to $\mathrm{EQE_{PV,CT}}(E)$ while the second term corresponds to $\mathrm{EQE_{PV,t}}(E)$. The fitting parameters for each material system are presented below.

Supplementary Table 1: Details of the Gaussian fits of the material systems presented in Fig.2.

Material System \ Fitting Parameters	E_{CT} (eV)	λ_{CT} (eV)	f_{CT} (eV^2)	E_t (eV)	λ_t (eV)	f_t (eV^2)	n
PM6:ITIC	1.61	0.15	9.0×10^{-2}	0.87	0.49	1.1×10^{-6}	1.85
PBDB-T:EH-IDTBR	1.62	0.17	5.9×10^{-3}	0.83	0.66	2.6×10^{-7}	1.93
BQR:$PC_{70}BM$	1.42	0.30	2.1×10^{-3}	0.75	0.60	3.1×10^{-7}	1.88
PBDB-T:ITIC	1.53	0.28	2.2×10^{-2}	0.87	0.45	2.9×10^{-7}	1.74
PTB7-Th:ITIC	1.44	0.47	9.0×10^{-2}	0.72	0.64	3.5×10^{-7}	1.99
PBDB-T:$PC_{70}BM$	1.39	0.56	2.5×10^{-2}	0.70	0.57	5.2×10^{-7}	1.96
PTB7-Th:$PC_{70}BM$	1.48	0.30	6.0×10^{-2}	0.82	0.32	3.1×10^{-7}	1.79
PCPDTBT:$PC_{70}BM$	1.33	0.29	1.6×10^{-2}	0.76	0.59	1.0×10^{-6}	1.74
PCDTBT:$PC_{70}BM$	1.48	0.35	4.9×10^{-3}	0.74	0.56	1.2×10^{-7}	2.02
PCDTBT:$PC_{70}BM$: 0.1% m-MTDATA	1.47	0.34	5.5×10^{-3}	0.72	0.60	3.8×10^{-7}	2.04
PCDTBT:$PC_{70}BM$:1% m-MTDATA	1.47	0.34	3.6×10^{-3}	0.85	0.50	1.5×10^{-6}	1.72

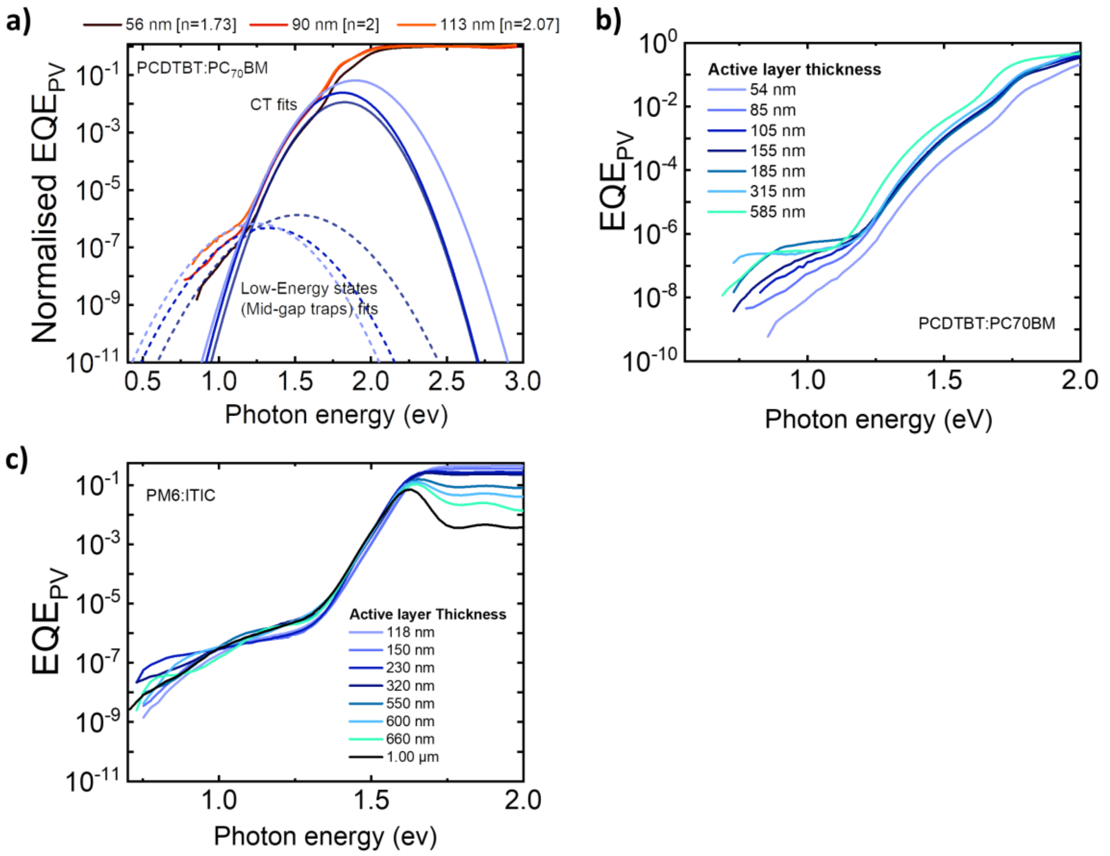

Supplementary Figure 2. **Thickness dependent EQE_{PV} (a)** The experimental EQE_{PV} of PCDTBT:PC_{70}BM solar cells *versus* photon energy with different thicknesses of the active layer. The fitting parameter *n* is affected by the thickness of the active layer. **(b)** and **(c)** are, respectively, the EQE_{PV} of PCDTBT:PC_{70}BM and PM6:ITIC devices with different active layer thicknesses showing the effect of optical interference on the shape of the EQE_{PV} in sub-gap region.

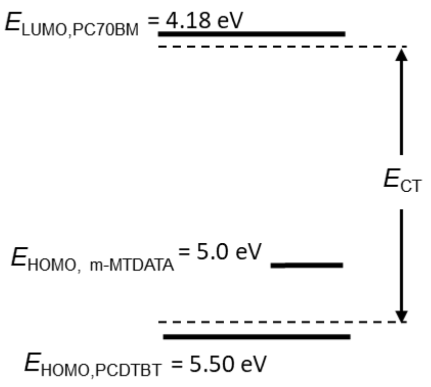

Supplementary Figure 3. **Energy level diagram:** The schematic energy level diagram of PCDTBT:$PC_{70}BM$ at the donor:acceptor interface and the HOMO level of m-MTDATA, functioning as a trap, is shown.

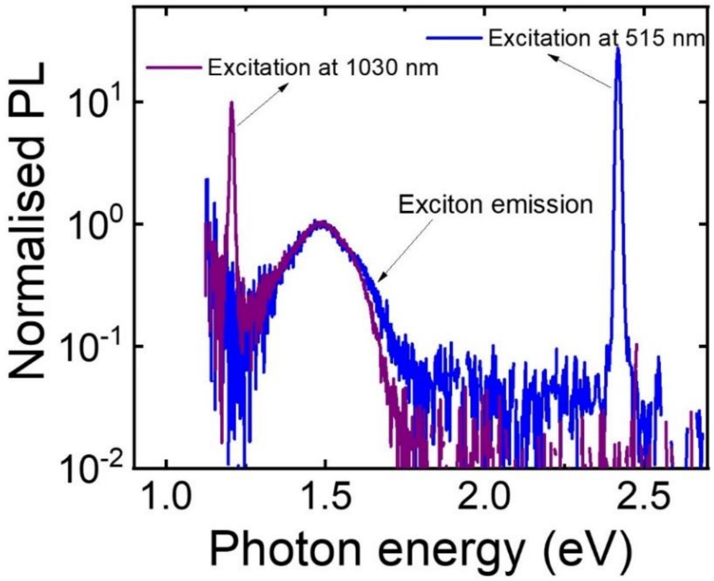

Supplementary Figure 4. **CT states PL spectrum.** The reduced photoluminescence (PL) of the PM6:ITIC blend, excited at 2.4 eV (515 nm), is plotted *versus* photon energy (eV). The PL of excited excitons (blue line) is mainly emitted at lower energies (CT states), while the shoulder at 1.4 eV corresponds to the PL of the lowest lying excitons. The PL of excited low-energy trap states (purple line), on the other hand, emits only at 1.5 eV (CT state energy) which is consistent with optical release and subsequent photon up-conversion.

Supplementary Note 2: Modified SRH Theory

The generation and recombination rates involving optical generation and radiative transitions of free electrons and holes taking place *via* trap states can be understood in terms of modified Shockley-Read-Hall (SRH) statistics.[1] After accounting for radiative transitions, the modified SRH net generation-recombination rate *via* traps reads

$$\mathcal{U}_{\mathrm{SRH}} = \frac{\tilde{c}_{\mathrm{n}}\tilde{c}_{\mathrm{p}}N_{\mathrm{t}}[np - n_1^{**}p_1^{**}]}{\tilde{c}_{\mathrm{n}}[n + n_1^{**}] + \tilde{c}_{\mathrm{p}}[p + p_1^{**}]} \qquad (2)$$

where n and p is the free electron and hole density, respectively, N_t is the trap density, while $\tilde{c}_{n(p)} = c_{n(p)} + r_{n(p)}$ is the coefficient for the transition of an electron (hole) between trap state and the conduction (valence) level being composed of radiative and non-radiative components $r_{n(p)}$ and $c_{n(p)}$, respectively. Here,

$$n_1^{**} = \frac{c_{\mathrm{n}}}{c_{\mathrm{n}} + r_{\mathrm{n}}}\left(n_1 + \frac{G_{\mathrm{n}}^{\mathrm{opt}}}{c_{\mathrm{n}}N_{\mathrm{t}}}\right) \qquad (3)$$

$$p_1^{**} = \frac{c_{\mathrm{p}}}{c_{\mathrm{p}} + r_{\mathrm{p}}}\left(p_1 + \frac{G_{\mathrm{p}}^{\mathrm{opt}}}{c_{\mathrm{p}}N_{\mathrm{t}}}\right) \qquad (4)$$

with $n_1 = N_{\mathrm{c}} \exp\left(\frac{[E_{\mathrm{t}} - E_{\mathrm{c}}]}{kT}\right)$ and $p_1 = N_{\mathrm{v}} \exp\left(\frac{[E_{\mathrm{v}} - E_{\mathrm{t}}]}{kT}\right)$, while $G_{\mathrm{n}}^{\mathrm{opt}}$ and $G_{\mathrm{p}}^{\mathrm{opt}}$ are the maximum optical generation rates for electrons and holes *via* traps, respectively, both depending linearly on the light intensity; E_{t} is the energy of the trap state, E_{c} is the energy of the conduction level, and E_{v} is the energy of the valence level. Note that the conduction and valance level coresepond to acceptor LUMO and donor HOMO levels, respectivly. In accordance with detailed balance, we furthermore have[2]

$$r_{\mathrm{n}} = \frac{1}{n_1}\int_0^{\infty} \sigma_{\mathrm{n}}^{\mathrm{opt}}(E)\Phi_{\mathrm{BB}}(E)dE \qquad (5)$$

$$r_{\mathrm{p}} = \frac{1}{p_1}\int_0^{\infty} \sigma_{\mathrm{p}}^{\mathrm{opt}}(E)\Phi_{\mathrm{BB}}(E)dE \qquad (6)$$

where $\sigma_{\mathrm{n(p)}}^{\mathrm{opt}}$ is the corresponding absorption cross section for electrons (holes) and Φ_{BB} is the black-body spectrum of the environment. Finally, the associated net recombination-generation current density *via* traps is given by

$$J_{\mathrm{SRH}} = q\int_0^d \mathcal{U}_{\mathrm{SRH}}\, dx \qquad (7)$$

where q is the elementray charge and d is the active layer thickness.

Derivation of the dark current density

For transitions predominately taking place *via* mid-gap states, we expect $n_1 = p_1 = n_i$ and $n \approx p \approx n_i \exp(qV/2kT)$, where n_i is the intrinsic carrier density. Then, assuming $\tilde{c}_{\mathrm{n}} = \tilde{c}_{\mathrm{p}} = \tilde{c}$ and that non-radiative transitions dominate over raditive ones (i.e. $c_{\mathrm{n(p)}} \gg r_{\mathrm{n(p)}}$), the associated current density in the dark ($G_{\mathrm{n}}^{\mathrm{opt}} = G_{\mathrm{p}}^{\mathrm{opt}} = 0$) simplifies as

$$J_{\mathrm{SRH}} = J_2 = J_{02}\left[\exp\left(\frac{qV}{2kT}\right) - 1\right] \qquad (8)$$

with $J_{02} = q\tilde{c}N_{\mathrm{t}}n_i d/2$ being the corresponding dark saturation current density. Furthermore, we assume optical transitions *via* mid-gap states to be governed by Marcus-type charge transfer with $\sigma_{\mathrm{n}}^{\mathrm{opt}} = \sigma_{\mathrm{p}}^{\mathrm{opt}} = \sigma_{\mathrm{t}}^{\mathrm{opt}}$. Accordingly, an absorption cross section of the form

$$\sigma_{\mathrm{t}}^{\mathrm{opt}}(E) = \frac{f_{\sigma\mathrm{t}}}{E\sqrt{4\pi\lambda_{\mathrm{t}}kT}}\exp\left(-\frac{[E_{\mathrm{t}} + \lambda_{\mathrm{t}} - E]^2}{4\lambda_{\mathrm{t}}kT}\right) \qquad (9)$$

is expected. Here, $f_{\sigma\mathrm{t}}$ is a prefactor that depends on the oscillator strength. On the other hand, the absorption coefficient for optical trap generation can be expressed as $\alpha_{\mathrm{t}} = \tilde{f}_{\mathrm{t}}\sigma_{\mathrm{t}}N_{\mathrm{t}}$, where $\tilde{f}_{\mathrm{t}}$ is the occupancy of the trap states which for mid-gap states is $\tilde{f}_{\mathrm{t}} \approx 1/2$. Then, after noting that for weakly absorbing states the EQE may be approximated as $\mathrm{EQE}_{\mathrm{PV,t}} = \alpha_{\mathrm{t}}d$, we finally obtain

$$J_{02} = \frac{q}{\mathrm{EQE}_{\mathrm{LED,t}}}\int_0^{\infty}\mathrm{EQE}_{\mathrm{PV,t}}(E)\Phi_{BB}(E)dE \qquad (10)$$

where $\mathrm{EQE}_{\mathrm{LED,t}} = r_{\mathrm{n(p)}}/\left[c_{\mathrm{n(p)}} + r_{\mathrm{n(p)}}\right]$ denotes the radiative efficiency of the states, while

$$\mathrm{EQE}_{\mathrm{PV,t}}(E) = \frac{f_t}{E\sqrt{4\pi\lambda_{\mathrm{t}}kT}}\exp\left(-\frac{[E_{\mathrm{t}} + \lambda_{\mathrm{t}} - E]^2}{4\lambda_{\mathrm{t}}kT}\right) \qquad (11)$$

with $f_{\mathrm{t}} = f_{\sigma\mathrm{t}}N_{\mathrm{t}}d/2$.

It should be noted that J_2 is generally dependent on the ligh intensity (*via* $G_{\mathrm{n}}^{\mathrm{opt}}$ and $G_{\mathrm{p}}^{\mathrm{opt}}$). However, owing to the extremely weak absorption of traps in our case, the rate $\mathcal{U}_{\mathrm{SRH}}$ is dominated by injected carriers in forward bias ($n \gg n_1^{**}$); hence, the expressions for J_2 and J_{20} derived for dark conditions remain valid under open-circuit conditions (at 1 sun).

Conditions when optical generation via mid-gap states dominates: $\mathrm{EQE}_{\mathrm{PV}}$ *vs PL*

The optical generation *via* traps becomes dominant under special conditions when the influence of injected carriers from the contacts is negligible and the generation of free charge carriers by direct optical transitions are absent. For mid-gap states, assuming thermal generation to be negligible ($G_{\mathrm{t}}^{\mathrm{opt}}\tau \gg n_{\mathrm{i}}$), the following simplified rate equation for free charge carriers can be obtained

$$\frac{n}{t_{\mathrm{col}}} = \frac{\left(G_{\mathrm{t}}^{\mathrm{opt}}\tau\right)^2 - n^2}{2\tau\left(n + G_{\mathrm{t}}^{\mathrm{opt}}\tau\right)} - \beta n^2 \qquad (12)$$

assuming $n = p$, $G_{\mathrm{n}}^{\mathrm{opt}} = G_{\mathrm{p}}^{\mathrm{opt}} = G_{\mathrm{t}}^{\mathrm{opt}}$ and $\tau = (\tilde{c}N_{\mathrm{t}})^{-1}$, where $\tilde{c} = \tilde{c}_{\mathrm{n}} = \tilde{c}_{\mathrm{p}} \gg r_{\mathrm{n}} = r_{\mathrm{p}}$; moreover, t_{col} is the charge collection time and β is the band to band recombination coefficient. Here, the term on the left-hand-side represents the charge extraction rate, while the first and second term on the right-hand-side corresponds to trap-assisted net generation-recombination rate (based on modified SRH theory) and the band to band recombination rate, respectively.

Under short-circuit conditions, the carrier density is expected to be small and the recombination terms negligible. Subsequently, the short-circuit current density, $J_{SC} \propto n/t_{col}$, takes the form

$$J_{\mathrm{SC}} \propto G_{\mathrm{t}}^{\mathrm{opt}} \qquad (13)$$

being linear with the light intensity. Hence, we expect the photocurrent induced by mid-gap states to be linear with light intensity *at short-circuit* (at low generation levels). This is also seen experimentally in Supplementry Figure 5.

In PL measurements, on the other hand, charge-extracting electrodes are absent, corresponding to $t_{col} = \infty$. Under these conditions, everything that is generated ultimately recombines; after neglecting third-order terms for the carrier density, we find

$$n^2 \approx \frac{\left(G_{\mathrm{t}}^{\mathrm{opt}}\tau\right)^2}{2\beta G_{\mathrm{t}}^{\mathrm{opt}}\tau^2 + 1} \qquad (14)$$

Subsequently, for PL originating from band to band recombination ($\mathrm{PL} \propto \beta n^2$), we expect a quadratic intensity dependence [$\mathrm{PL} \propto \left(G_{\mathrm{t}}^{\mathrm{opt}}\right)^2$] at low intensities and a linear intensity dependence ($\mathrm{PL} \propto G_{\mathrm{t}}^{\mathrm{opt}}$) at high intensities. This explains the experimentally observed behavior in Supplementry Figure 6.

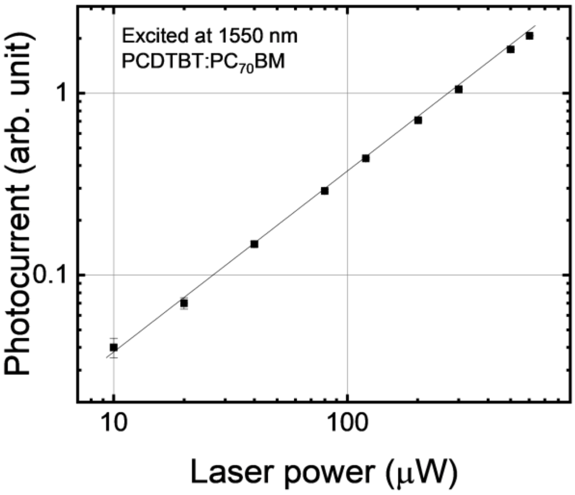

Supplementary Figure 5. Intensity-dependent photocurrent measurement: The photocurrent *versus* intensity measurement at excitation wavelength of 1550 nm for a PCDTBT:$PC_{70}BM$ device at short-circuit. The photocurrent is in this case exclusively induced by optical generation *via* mid-gap states, showing a linear intensity dependence, as expected from modified SRH theory.

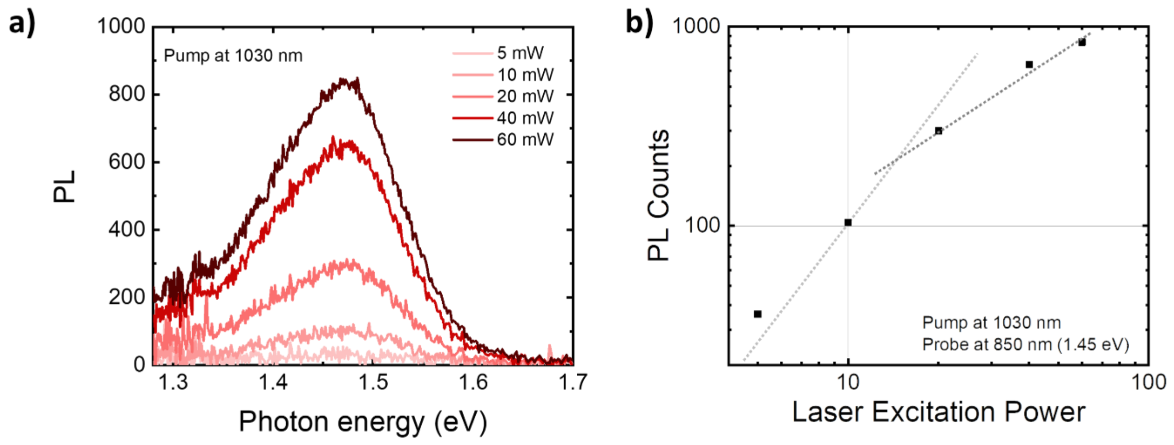

Supplementary Figure 6. **Intensity dependent PL measurement. (a)** PL spectra measured with different pump intensities plotted *versus* photon energy. **(b)** PL spectra count at the peak (1.45 eV) is plotted *versus* laser excitation power. The increase of the laser power leads to a quadratic growth of the PL intensity at lower power, while a linear dependence is observed at higher power. This behaviour is consistent with the behaviour expected from modified SRH theory (see above), strongly supporting the presence of optical release.

Supplementary Note 3: The Two-Diode model

The total bulk recombination current, taking place *via* CT and the low-energy sub-gap channels, is described by two parallel currents J_1 and J_2 being the CT-induced recombination current and the trap-induced recombination current, respectively. The total dark current is given by

$$J_{tot} = J_1 + J_2 + J_{\text{shunt}} \qquad (15)$$

where J_{shunt} is the additional leakage current induced by an external shunt resistance (caused by non-idealities in the device fabrication). This system can be described in terms of the equivalent circuit shown in Supplementary Figure 7.

Accordingly, the diode current J_1 only involves the direct recombination between free electrons and holes, being governed by their respective quasi-Fermi levels (E_{Fn} for electrons, and E_{Fp} for holes). The associated current-voltage (*J*-*V*) characteristics is governed by the quasi-Fermi level difference $qV = E_{\text{Fn}} - E_{\text{Fp}}$ between electrons and holes [2] (at the electrodes); hence,

$$J_1 = J_{01}\left[\exp\left(\frac{qV}{kT}\right) - 1\right] \qquad (16)$$

where J_{01} is the corresponding dark saturation current which is given by

$$J_{01} = \frac{q}{\text{EQE}_{\text{LED,CT}}}\int_0^{\infty} \text{EQE}_{\text{PV,CT}}\Phi_{\text{BB}}\, dE \qquad (17)$$

in accordance with the reciprocity principle; Φ_{BB} is the black-body spectrum at $T = 300\text{K}$ (k is the Boltzmann constant and T is the absolute temperature).

On the other hand, the recombination (and dark generation) of free electrons and holes *via* trap states is composed of a two-step process: (i) the transition involving a free hole and a trap, and (ii) the transition involving a free electron and a trap. Furthermore, in accordance with Shockley-Read-Hall statistics,[2] trapped carriers occupying the mid-gap states can be described by their own quasi-fermi level E_{Ft}. Subsequently, the diode current J_2 induced by trap-assisted recombination between free electrons and holes can be described by two diode components which are ***in series*** with each other: the first diode current J_1^t being governed by the quasi-Fermi level difference $qV_1^{\text{t}} = E_{\text{Ft}} - E_{\text{Fp}}$ (i), and the second J_2^t by $qV_2^t = E_{\text{Fn}} - E_{\text{Ft}}$ (ii); hence,

$$J_1^{\text{t}} = J_{01}^{\text{t}}\left[\exp\left(\frac{qV_1^{\text{t}}}{kT}\right) - 1\right] \qquad (18)$$

$$J_2^t = J_{02}^t\left[\exp\left(\frac{qV_2^{\text{t}}}{kT}\right) - 1\right] \qquad (19)$$

where J_{01}^t and J_{02}^t are the corresponding dark saturation currents associated with process (i) and (ii), respectively. For the case when mid-gap traps are dominant, we expect $V_1^{\text{t}} = V_2^{\text{t}} = V/2$. Then, noting that $J_2 = J_1^{\text{t}} = J_2^{\text{t}}$ (because of the series connection), it follows that

$$J_2 = J_{02}\left[\exp\left(\frac{qV}{2kT}\right) - 1\right] \qquad (20)$$

with J_{02} being the corresponding dark saturation current for this recombination channel, which in general is composed both radiative and non-radiative transitions. An explicit expression for

J_{02} can be derived based on the modified SRH theory which takes radiative transitions *via* traps into account (see Supplementary Note 2). We find

$$J_{02} = \frac{q}{\mathrm{EQE}_{\mathrm{LED,t}}} \int_{0}^{\infty} \mathrm{EQE}_{\mathrm{PV,t}} \Phi_{\mathrm{BB}} \, dE \qquad (21)$$

where $\mathrm{EQE}_{\mathrm{LED,t}}$ and $\mathrm{EQE}_{\mathrm{PV,\,t}}$ are the electroluminescent and photovoltaic external quantum efficiencies associated with recombination and absorption of mid-gap states, respectively.

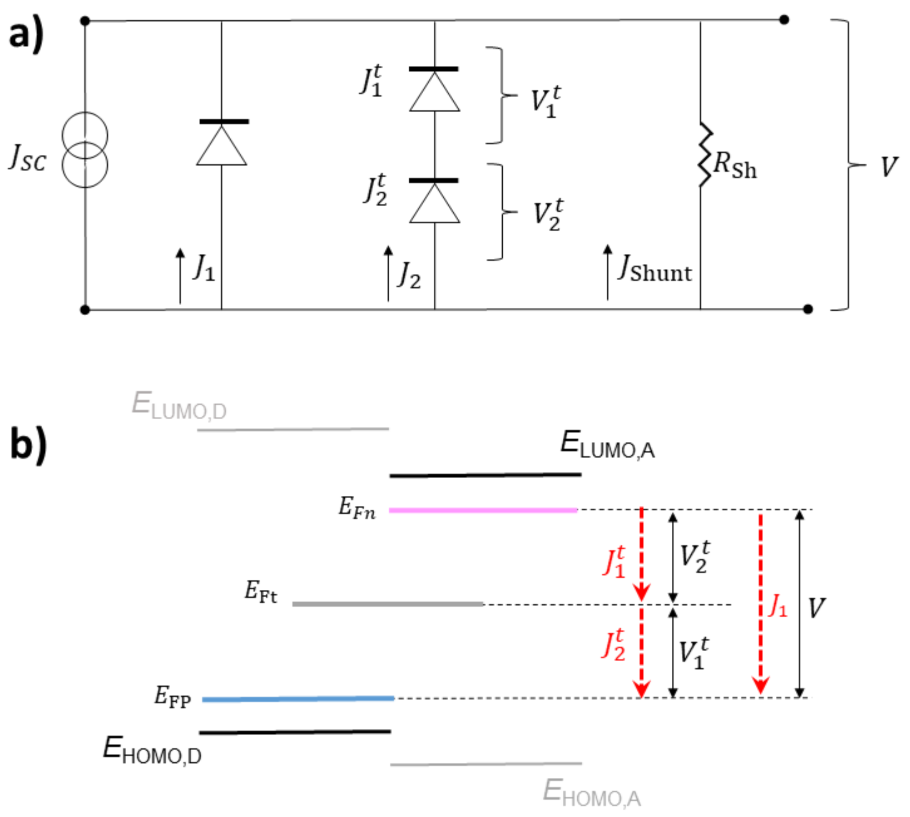

Supplementary Figure 7. **Two-diode model for describing the dark *J*-*V* characteristics.** **(a)** The equivalent circuit of the assumed two-diode model. Two recombination current J_1 and J_2 are shown in the circuit, where J_2 is described by two diodes which are in series with each other. **(b)** The schematic energy levels at the donor-acceptor interface are shown together with the quasi-Fermi levels of electrons (E_{Fn}), holes (E_{Fp}) and the traps (E_{Ft}). The band to band recombination current J_1 and the trap assisted recombination currents J_1^{t} and J_2^{t} are shown with downwards arrows.

Supplementary Table 2. Details of the two diode model fits.

Material System \ Fitting Parameters	R_{Shunt} ($\Omega.cm^2$)	$EQE_{LED,CT}$	$EQE_{LED,t}$
PM6:Y6	1.8×10^6	5.9×10^{-5}	4.1×10^{-8}
BQR:PC_{70}BM	7.4×10^5	8.2×10^{-6}	9.3×10^{-5}

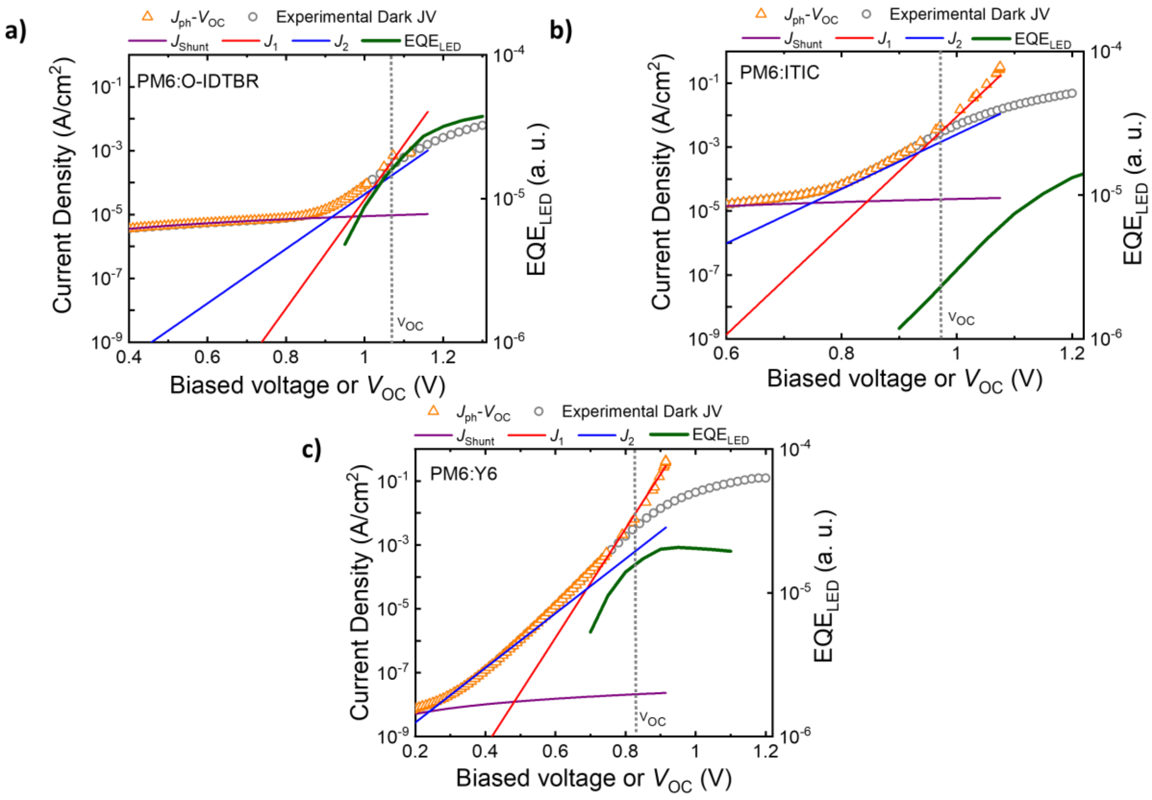

Supplementary Figure 8. Current and EQE_{LED} *versus* Voltage in donor acceptor systems with different E_{CT}: Two-diode fittings are performed on three exemplary organic solar cells **(a).** PM6:O-IDTBR **(b)** PM6:ITIC and **(C)** PM6:Y6. The V_{OC} is marked with a vertical dashed line on the plots. In all three systems the EQE_{LED} (green curves) at lower voltages is clearly voltage dependent due to the trade-off between radiative J_1 (red curve) and non-radiative J_2 (blue curve). In PM6:Y6 the variations in the EQE_{LED} near V_{OC} is marginal where J_1 dominates the current. In PM6:O-IDTBR and PM6:ITIC J_1 and J_2 are approximately equally contributing to the dark current with the EQE_{LED} being significantly voltage dependent.

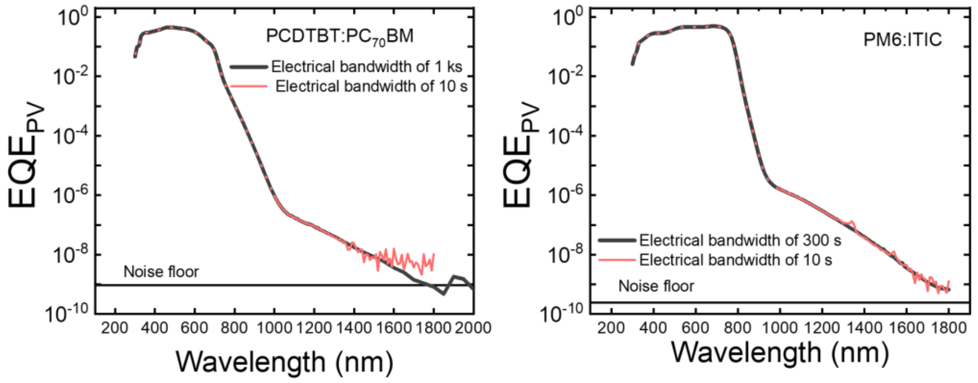

Supplementary Figure 9. **Noise floor of the EQE_{PV} measurement for two exemplary systems.** EQE_{PV} measured with different electrical bandwidths. The noise floor of the EQE_{PV} is shown by the horizontal line and is dependent on the thermal noise of the current defined by the shunt resistance of the device.

Supplemmentry References

1 Beaucarne, G., Brown, A., Keevers, M., Corkish, R. & Green, M. The impurity photovoltaic (IPV) effect in wide-bandgap semiconductors: an opportunity for very-high-efficiency solar cells? *Prog. Photovolt: Res. Appl.* **10**, 345-353 (2002).

2 Würfel, P. & Würfel, U. *Physics of solar cells: from basic principles to advanced concepts*. (John Wiley & Sons, 2016).
